# Blinatumomab demonstrates MRD eradication in MRD-positive/chemotherapy-delayed pediatric B-ALL and high response in relapsed/refractory cases: a multicenter cohort study

**DOI:** 10.3389/fimmu.2025.1607138

**Published:** 2025-09-18

**Authors:** Na Zhang, Wenting Hu, Yunpeng Dai, Jian Wang, Lijun Qu, Dan Wang, Bingju Liu, Jingbo Shao, Shuhong Shen, Hui Jiang

**Affiliations:** ^1^ Department of Hematology and Oncology, Shanghai Children’s Hospital, School of Medicine, Shanghai Jiao Tong University, Shanghai, China; ^2^ Department of Hematology and Oncology, Shanghai Children’s Medical Center, School of Medicine, Shanghai Jiao Tong University, Shanghai, China; ^3^ Department of Pediatric Hematology and Oncology, Shandong Provincial Hospital Affiliated to Shandong First Medical University, Jinan, China; ^4^ Department of Hematology and Oncology, Anhui Children’s Hospital, Hefei, China

**Keywords:** blinatumomab, B-cell acute lymphoblastic leukemia, children, minimal residual disease, relapsed/refractory, T cell activation

## Abstract

**Background:**

Blinatumomab, a bispecific T-cell engager targeting CD3+ and CD19+, promotes T cell–mediated cytotoxicity against B-cell precursor acute lymphoblastic leukemia (B-ALL). While its efficacy is established in relapsed/refractory (R/R) disease, its role as preemptive therapy for minimal residual disease (MRD)–positive patients or those experiencing chemotherapy delays remains undefined. Predictors of treatment failure also require further investigation.

**Methods:**

In this multicenter retrospective study, 105 patients who received blinatumomab were enrolled. Of these, 30 had R/R ALL, 21 were in complete remission (CR) with MRD positivity (CR-MRD^pos^), and 54 experienced chemotherapy delays. Eight patients received blinatumomab directly as reinduction therapy and 22 patients received burden-reduction chemotherapy prior to blinatumomab. In total, 11 children were in R/R status and 40 were in CR-MRD^pos^ before treatment. Patients were subsequently bridged to stem cell transplantation, chimeric antigen receptor T-cell therapy (CAR-T), or protocol continuation. Treatment response was analyzed across CR-MRD^pos^, R/R, and CR with MRD negativity (CR-MRD^neg^). Immune reconstitution profiles (T-cell subsets, cytokine dynamics), cytogenetic markers, and clinical outcomes were assessed to identify predictors of treatment resistance.

**Results:**

The CR rate was 81.8% in R/R and 82.5% in CR-MRD^pos^ patients (P = 1.000). Of 74 courses with CR-MRD^neg^, 73 remained MRD-negative during treatment. Univariate analysis revealed poor cytogenetics (P = 0.0001), CD19+ B-cell loss (P = 0.046), and BCR-ABL1 positivity (P = 0.002) as predictors of poor response. Cox regression analysis identified high MRD (P = 0.014), BCR/ABL1 (P = 0.065), and poor cytogenetics (P = 0.025) as independent risk factors. Blinatumomab significantly increased CD3+ T cells [0.96 (0.03–3.79) to 1.13 (0.26–7.74) ×10^9^/L, P = 0.016], along with CD4+ [0.35 (0.01–1.39) to 0.47 (0.07–2.94) ×10^9^/L] and CD8+ T cells [0.41 (0.01–2.39) to 0.56 (0.07–6.07) ×10^9^/L] (P = 0.005 and P = 0.006, respectively).The 1-year event-free survival for CR-MRD^neg^, CR-MRD^pos^, and R/R patients was 97.8% ± 2.2%, 86.7% ± 6.2%, and 73.3% ± 8.1%, respectively (P = 0.001), while overall survival was 97.8% ± 2.2%, 100%, and 93.3% ± 4.6% (P = 0.029).

**Conclusions:**

Blinatumomab effectively clears MRD as preemptive therapy and serves as a bridging strategy during chemotherapy delays in pediatric B-ALL, while maintaining high response rates in R/R cases.

## Introduction

Survival rates for B-cell acute lymphoblastic leukemia (B-ALL) have significantly improved in recent years. However, approximately 15% of children with B-ALL experience relapse after frontline chemotherapy (1). Based on the site and timing of relapses, these children are classified as having standard- or high-risk first-relapse B-ALL (2).

Blinatumomab is a bispecific T-cell–engaging antibody that binds CD3+ T cells and CD19+ leukemia cells, inducing cytotoxic immune responses that lyse CD19-expressing B cells via activated T cells (3). Meta-analyses have demonstrated the potent therapeutic efficacy and favorable safety profile of blinatumomab in children with relapsed/refractory (R/R) B-ALL (4). It has also induced high rates of complete minimal residual disease (MRD) response in both adults and children with molecularly resistant B-ALL.

Nevertheless, 10%–15% of patients exhibit primary resistance to blinatumomab. Emerging evidence has identified several mechanisms contributing to treatment failure. Elevated levels of regulatory T cells, characterized by CD4/CD25/FOXP3 expression and interleukin-10 (IL-10)–mediated suppression of T-cell proliferation, have been associated with reduced response (5). Increased expression of programmed death ligand 1 (PD-L1), the binding ligand of the inhibitory checkpoint molecule programmed death 1 (PD-1), has also been linked to impaired T-cell function and diminished efficacy (6). Additionally, KMT2A-rearranged ALL lineage switch may induce resistance (7, 8). Lower blast counts in bone marrow (BM) (≤50%) have been associated with better response than higher disease burden (9, 10).

Despite these insights, the mechanisms underlying blinatumomab resistance remain incompletely elucidated. With the expanding application of blinatumomab as frontline preemptive therapy, particularly for patients with persistent complete remission with MRD positivity (CR-MRD^pos^) or chemotherapy delays, its therapeutic scope has broadened. In this study, we comprehensively analyzed treatment response across three cohorts: CR-MRD^pos^, R/R, and CR with MRD negativity (CR-MRD^neg^). Through systematic evaluation of immune reconstitution profiles (T-cell subsets, cytokine dynamics), cytogenetic markers, and clinical outcomes, we aimed to identify key predictors of treatment resistance.

## Methods

### Patients

This multicenter retrospective study was approved by the institutional review boards (No. 2025R022-E01) following discussion in a multicenter advisory panel across four pediatric medical centers: Shanghai Children’s Hospital, Shanghai Children’s Medical Center, Anhui Children’s Hospital, and Shandong Provincial Hospital. Patients ≤18 years old with R/R B-ALL and MRD positivity at any time who received blinatumomab therapy were enrolled. Patients with chemotherapy intolerance or severe infection who received blinatumomab as bridging therapy were enrolled as the control group. The exclusion criteria were as follows: (i) patients with severe infection and cardiac, liver, or kidney insufficiency who had an expected survival time of less than 3 months; and (ii) those who received blinatumomab for fewer than 7 days. Patient enrollment lasted from September 2021 to June 2024, with follow-up through March 2025. A total of 105 patients were enrolled.

### Treatment strategy

Blinatumomab was administered via a stepwise dose-escalation protocol during the initial cycle: 5 μg/m²/day as continuous intravenous infusion on days 2–7, followed by escalation to 15 μg/m²/day for a total cycle duration of 14–28 days. The infusion duration depended on family financial conditions and physician discretion. In some cases, BM aspiration was performed on day 15, and treatment was discontinued upon achieving BM remission. Each treatment cycle was separated by a 14-day treatment break.

Dexamethasone prophylaxis (5 mg/m²/day for 1 day) was routinely administered. Subsequent treatment cycles commenced directly at 15 μg/m²/day. Adverse events (AEs) were managed according to the manufacturer’s instructions. BM assessment was performed upon completion of the infusion cycle. Intrathecal injections of methotrexate, cytarabine, and dexamethasone were administered before, during, or after blinatumomab cycles. Upon achieving BM remission, patients proceeded to hematopoietic stem cell transplantation (HSCT), continued the original protocol, or received alternative treatment. Non-responders were transitioned to salvage protocols, chimeric antigen receptor T-cell (CAR-T) therapy, or palliative HSCT, as clinically indicated.

Some patients received reinduction therapy to reduce tumor burden. The reinduction therapy followed the initial induction regimen: dexamethasone 6 mg/m² on days 1–4; vincristine 1.5 mg/m² on days 5, 12, 19, and 26; prednisone 45 mg/m² on days 5–28; daunorubicin 25 mg/m² on days 5 and 12; and peg-asparaginase 2,000 U/m² on days 6 and 26.

For patients with Philadelphia chromosome–positive (Ph+) disease, a tyrosine kinase inhibitor (TKI) was added, with dasatinib 80 mg/m² preferred. Bridging chemotherapy prior to blinatumomab included induction chemotherapy or continued consolidation chemotherapy consisting of cyclophosphamide 1,000 mg/m² on day 1, cytarabine 50 mg/m² on days 1–7, and mercaptopurine 40 mg/m² on days 1–7.

### Definition

Patients were divided into three groups: CR-MRD^neg^, CR-MRD^pos^, and R/R. Response was categorized as either cytological CR or MRD CR. Cytological CR was defined as <5% BM blasts in patients with R/R status. MRD CR, detected by flow cytometry (FCM), was defined as a reduction in MRD to <0.01% or maintenance of MRD negativity in patients with CR-MRD^pos^. No response was defined as partial remission (PR) or no remission (NR) in R/R patients, and persistent MRD ≥0.01% in patients with CR-MRD^pos^.

Poor cytogenetics were defined as KMT2Ar, BCR*-*ABL1, and TCF3*-*HLF, according to the Chinese Children’s Cancer Group ALL (CCCG-ALL) 2015 protocol. Event-free survival (EFS) was defined as the time from diagnosis to relapse, death, secondary cancer, or last contact for those who were event-free. Overall survival (OS) was defined as the time from diagnosis to death from any cause or last contact if alive.

### Cytokine detection

Serum concentrations of target cytokines [IL-1β, IL-2, IL-4, IL-5, IL-6, IL-8, IL-10, IL-12p70, IL-17, interferon (IFN)-γ, tumor necrosis factor (TNF)-α, and IFN-α] were measured using a multiplex microsphere-based flow immunofluorescence assay (12-cytokine kits, Raisecare, China) according to the manufacturer’s instructions. Cytokines were assessed before blinatumomab infusion, at the onset of cytokine release syndrome (CRS) or immune effector cell–associated neurotoxicity syndrome (ICANS), and at the end of blinatumomab treatment.

### T- cell and B-cell subsets

Basic lymphocyte subpopulations were analyzed using a FACSCalibur flow cytometer (BD Biosciences) and reported as both percentages and absolute counts. FCM with CellQuest software (BD Biosciences) was used for analysis of lymphocyte subsets (CD3/CD45/CD4/CD8/CD16CD56/CD19, BD Biosciences), including T cells (CD3+CD45+), cytotoxic T cells (CD3+CD8+CD45+), helper T cells (CD3+CD4+CD45+), NK cells (CD16+CD56+CD3−CD45+), and B cells (CD19+CD45+). A total of 15,000 lymphocytes were acquired for analysis. Data were collected at baseline and on days 14, 21, and 28 (end of treatment). T-cell activation magnitude was defined as the difference between post-blinatumomab and baseline (pre-treatment) measurements.

### Statistical analysis

Quantitative data with a Gaussian distribution were presented as mean ± standard deviation (SD) and compared using the t-test. Non-normally distributed data were presented as medians with full ranges. Comparisons between two groups were performed using the Mann–Whitney U test or Wilcoxon matched-pairs test. Comparisons involving two or more factors were conducted using one-way or two-way analysis of variance (ANOVA). Categorical variables were presented as percentages and compared using Fisher’s exact test or the chi-square (χ²) test. Patients lost to follow-up were censored at the last date they were known to be alive. OS and EFS were estimated by the Kaplan–Meier method, and curves were compared using the log-rank test. The Cox proportional hazards model was used for univariate and multivariate analyses. All statistical analyses were performed using GraphPad Prism version 9 (GraphPad Software Inc., La Jolla, CA, USA) and SPSS version 19.0 (SPSS Inc., Chicago, IL, USA). All tests were two-sided, and P < 0.05 was considered statistically significant.

## Results

### Patients’ characteristics

A total of 105 patients with B-ALL received blinatumomab across 125 cycles, following standardized clinical practice. The median age was 72 months (range, 5–210 months). Sixty-four patients (61%) were male and 41 (39%) were female.

Thirty cases were diagnosed with R/R ALL, including 23 with relapse and seven with induction failure. Eight patients received blinatumomab directly as reinduction therapy, and 22 patients received burden-reduction chemotherapy prior to blinatumomab. Among these, three patients failed to reduce tumor burden, with blast levels remaining above 5%.

Ten patients achieved CR-MRD^pos^, and nine patients achieved CR-MRD^neg^. An additional 21 patients had CR-MRD^pos^ following induction or developed MRD positivity during treatment. Fifty-four patients received blinatumomab due to chemotherapy delay caused by chemotherapy intolerance or severe AEs, among whom nine patients had CR-MRD^pos^.

At the initial cycle of blinatumomab, 40 patients had CR-MRD^pos^, 11 patients had R/R status, and 54 patients had MRD negativity. Among the latter, four cases had BM blasts ranging from 5%–9.5%, while seven had blasts ≥20%. For these 51 patients, the median BM blast percentage was 2% and the median MRD percentage was 0.52% (**Table 1**). In patients achieving MRD negativity, a total of 74 cycles were administered. The demographic and clinical characteristics of the R/R, CR-MRD^pos^, and CR-MRD^neg^ groups are summarized in **Table 1**.

**Table 1:** Patient characteristics of those treated with blinatumomab.

Characteristics	All patients	R/r, CR-MRD^pos^	CR-MRD^neg^	P value
Numbers, n	105	51	54	–
Gender (M/F), n	64/41	34/17	30/24	0.317
Age, median (range), mons	72 (5-210)	69 (5-208)	73.5 (5-210)	0.593
BM blasts, median (range), %	2 (0-80)	2 (0-80)	–	–
BM MRD, median (range), %	0.52 (0.004-63)	0.52 (0.004-63)	–	–
Cytogenetic characteristics				
t(9;22), BCR/ABL1, n (%)	12 (11.4)	5 (9.8)	7 (13.0)	0.762
t(v;11q23), KMT2Ar, n (%)	11 (10.5)	7 (13.7)	4 (7.4)	0.350
TCF3/HLF, n (%)	1 (1.0)	1 (2.0)	0	0.486
t(12;21), ETV6/RUNX1, n (%)	21 (20.0)	5 (9.8)	16 (29.6)	0.014
E2A/PBX1, n (%)	4 (3.8)	2 (3.9)	2 (3.7)	1.000
ZNF384r, n (%)	5 (4.8)	2 (3.9)	3 (5.6)	1.000
PAX5r, n (%)	2 (1.9)	1 (2.0)	1(1.9)	1.000

MRD, minimal residual disease; R/R, relapsed/refractory; CR-MRD^pos^, complete remission with MRD positive; CR-MRD^neg^, complete remission with MRD negative; BM, bone marrow; M/F, male/female.

### Response rate

The CR rate of R/R patients was 81.8% (9/11), while the MRD-negative CR rate was 72.7% (8/11). MRD CR was achieved in 33 of 40 cases (82.5%) with CR-MRD^pos^. The overall CR response rate was 82.4%.

Among the R/R and CR-MRD^pos^ patients, 31 received a 14-day infusion and 20 received a 3–4-week infusion. The response rate was 83.9% for the 2-week regimen and 80% for the 3–4-week regimen.

Of the 74 MRD-negative cycles, 73 patients remained MRD negative until the follow-up day. Only one patient experienced central nervous system (CNS) relapse 14 months after blinatumomab.

In nine cases with NR after the first blinatumomab cycle, three received a second cycle of blinatumomab, two patients underwent HSCT (achieving CR), three patients with persistent MRD continued chemotherapy (achieving CR), and one patient discontinued treatment and subsequently died. Among the three patients receiving a second cycle of blinatumomab, one achieved CR and bridged to HSCT. The remaining two patients failed to achieve remission and continued treatment with CAR-T. All three patients remained alive.

### T-cell response after blinatumomab

#### CD3+ T cell activation

We compared data obtained before and after 2–4 weeks of blinatumomab infusion. The data exhibited a non-Gaussian distribution and were expressed as median (range). Comparisons were performed using the Wilcoxon matched-pairs test. Detailed data are shown in **Supplementary Table S1**.

**Figure 1:**
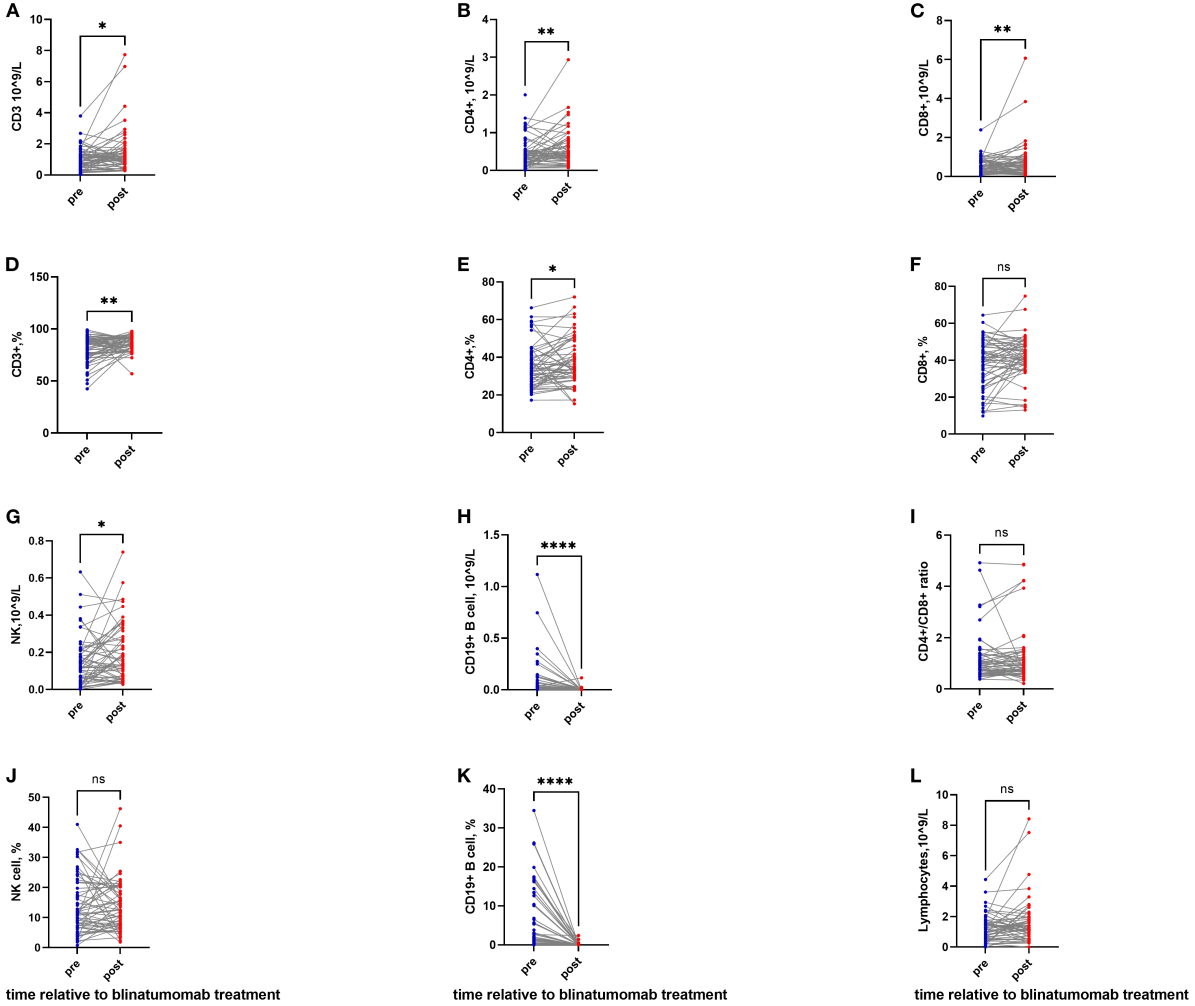
T-cell activation and B-cell depletion following blinatumomab therapy. **(A, D)** The absolute count and percentage of CD3+ T cells significantly increased after blinatumomab. **(B, E)** The absolute count and percentage of CD4+ T cells significantly increased after blinatumomab. **(C, F)** The absolute count of CD8+ T cells elevated significantly, while the percentage remained stable. **(G, J)** NK cell counts showed a significant increase, though the percentage remained unchanged. **(H, K)** B cells were depleted profoundly in both absolute and percentage. **(I)** The CD4+/CD8+ ratio exhibited a non-significant decrease. **(L)** Total lymphocyte count remained stable throughout treatment. *P< 0.05; **P< 0.01; ****P< 0.0001.

The absolute count of CD3+ T cells significantly increased from 0.96 (0.03–3.79) × 10^9^/L to 1.13 (0.26–7.74) ×10^9^/L (P = 0.016; **Figure 1A**). The CD3+ percentage also rose significantly (P = 0.008; **Supplementary Table S1**, **Figure 1D**).

CD4+ and CD8+ T cells also increased, from 0.35 (0.01–1.39) to 0.47 (0.07–2.94) ×10^9^/L, and from 0.41 (0.01–2.39) to 0.56 (0.07–6.07) ×10^9^/L, respectively (P = 0.005 and *P* = 0.006; **Figures 1B, C**). The percentages of CD4+ and CD8+ T cells also increased (P = 0.025 and *P* = 0.054; **Supplementary Table S1**, **Figures 1E, F**).

The CD4/CD8 ratio exhibited a nonsignificant decrease from 0.85 (0.37–4.92) to 0.78 (0.21–4.84) (P = 0.532; **Figure 1I**). The CD16+CD56+/CD3− NK cell count increased from 0.12 (0.00–0.63) ×10^9^/L to 0.15 (0.03–0.74) ×10^9^/L (P = 0.024; **Figure 1G**), although the percentage remained unchanged (**Figure 1J**).

B cells were completely depleted, with absolute counts decreasing from 0.007×10^9^/L (0.00–1.12) to undetectable levels 0.00×10^9^/L (0.00–0.11) (P <0.0001; **Figure 1H**), and percentages falling from 1.39% (0.00–34.48) to 0% (0.00–2.43) (P < 0.0001; **Figure 1K**). Total lymphocyte counts remained stable throughout the observation period.

In patients receiving a 14-day infusion, CD3+, CD4+, and CD8+ T cells had already increased compared with baseline (**Supplementary Table S1**). A subset of NK cells also expanded significantly, from 0.12 (0.00–0.63) to 0.18 (0.03–0.74) ×10^9^/L (P = 0.013).

In patients receiving a 21/28-day infusion, CD3+ T cells continued to increase, rising from 0.80 (0.03–1.86) to 1.01 (0.33–7.74) ×10^9^/L (P = 0.033; **Supplementary Table S1**). CD4+ T cells showed a sustained elevation from 0.42 (0.01–0.72) to 0.47 (0.12–1.25) ×10^9^/L (P = 0.092), while CD8+ and NK cells declined (P = 0.470 and *P* = 0.850; **Supplementary Table S1**).

Levels of Immunoglobulin (Ig)

Levels of G, IgA, and IgM were assessed before and after therapy. Following blinatumomab, IgG levels went down from 9.23 (2.28–18.50) to 7.05 (1.61–18.00) g/L (P = 0.0005). IgA levels and IgM levels also declined from 0.78 (0.12–2.39) to 0.27 (0.03–0.92) g/L (P < 0.0001) and from 0.41 (0.11-1.31) to 0.19 (0.01- 0.56) g/L, respectively (both P < 0.0001).

### CD3+ T cell activation in MRD^pos^+R/R and MRD^neg^ patients

When patients with R/R and CR-MRD^pos^ status were compared with those with CR-MRD^neg^, notable differences in the immune cell repertoire were observed. The data exhibited a non-Gaussian distribution and were expressed as median (range), with comparisons performed using the Mann–Whitney U test.

**Figure 2:**
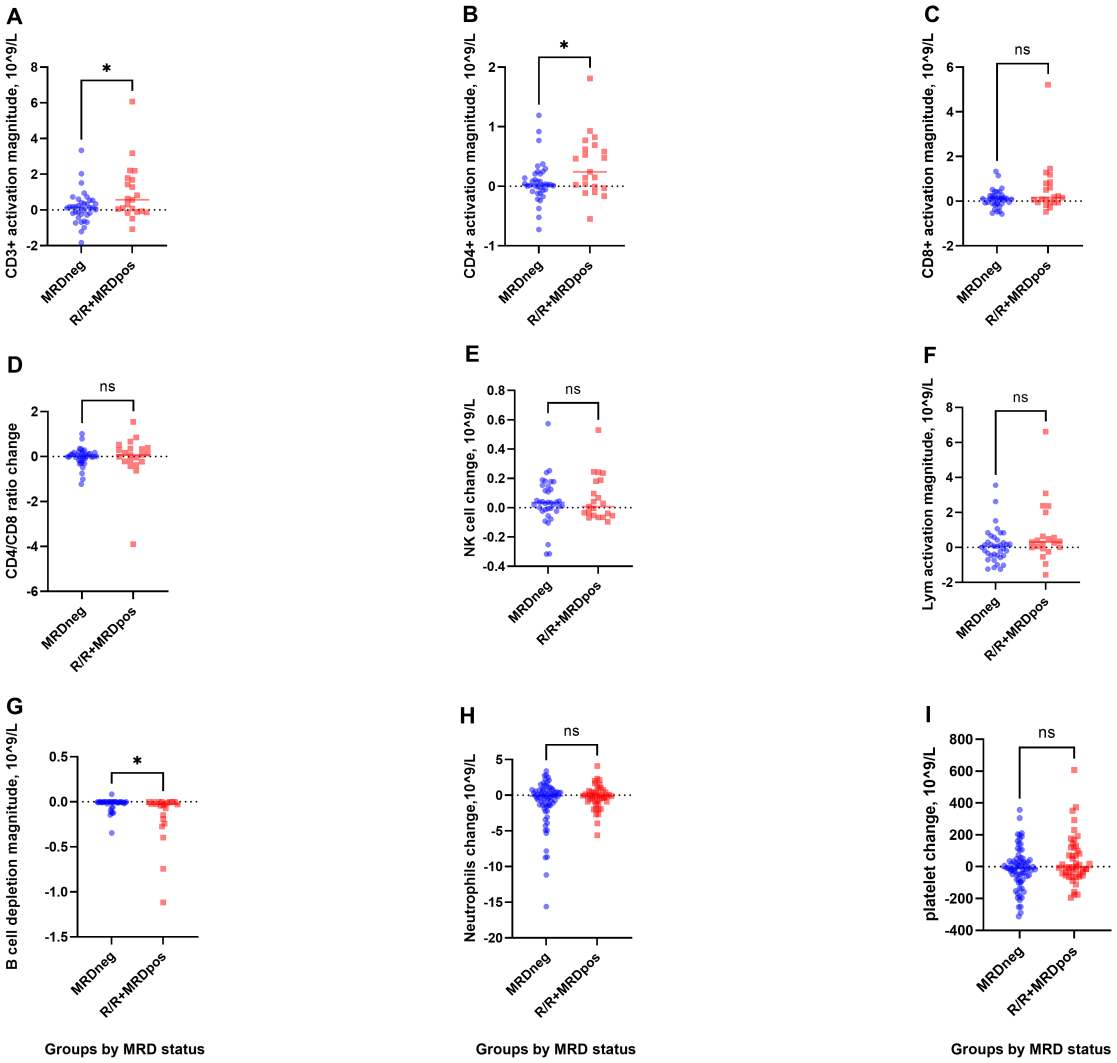
T-cell activation and B-cell depletion in R/R+MRD^pos^ and CR-MRD^neg^ groups. **(A, B)**, Greater increases in CD3+ and CD4+ T-cell counts were observed in the R/R+MRD^pos^ cohort. **(C-F)**, CD8+, CD4+/CD8+ ratio, NK cells, and lymphocytes showed mild fluctuations. **(G)**, Enhanced B-cell depletion was observed in R/R+MRD^pos^ patients. **(H, I)**, No significant intergroup differences were observed in neutrophils or platelets. *P< 0.05.

The increase in CD3+ T cells was significantly greater in the R/R + CR-MRDpos group than in the CR-MRD^neg^ group [0.57 (−1.08 to 6.07) vs. 0.12 (-1.83 to 3.34)×10^9^/L; P = 0.047; **Figure 2A**]. CD4+ cells expanded more significantly in the R/R + CR-MRDpos group than in the CR-MRD^neg^ group [0.24 (−0.55 to 1.81) *vs*. 0.03 (−0.73 to 1.19) × 10^9/L; P = 0.039; **Figure 2B**]. In contrast, no significant difference in CD8+ T-cell expansion was observed between the two cohorts [0.16 (−0.48 to 5.21) *vs*. 0.11 (−0.58 to 1.33) × 10^9/L; P = 0.174; **Figure 2C**].

Enhanced B-cell eradication was observed in the R/R + CR-MRD^pos^ group compared with the CR-MRD^neg^ group [−0.03 (−1.12 to 0.00) *vs*. −0.004 (−0.35 to 0.08)×10^9^/L, P = 0.017; **Figure 2G**]. No significant intergroup differences were detected in NK cells, total lymphocytes, CD4+/CD8+ ratio, neutrophils, or platelets (**Figures 2D–F, H, I**; **Supplementary Table S2**).

### Cytokine level following CRS

At the onset of CRS, significant elevations in serum levels of IL-2, IL-5, IL-10, and IFN-γ were observed. The data were expressed as median (range), and comparisons were performed using the Wilcoxon matched-pairs test.

**Figure 3:**
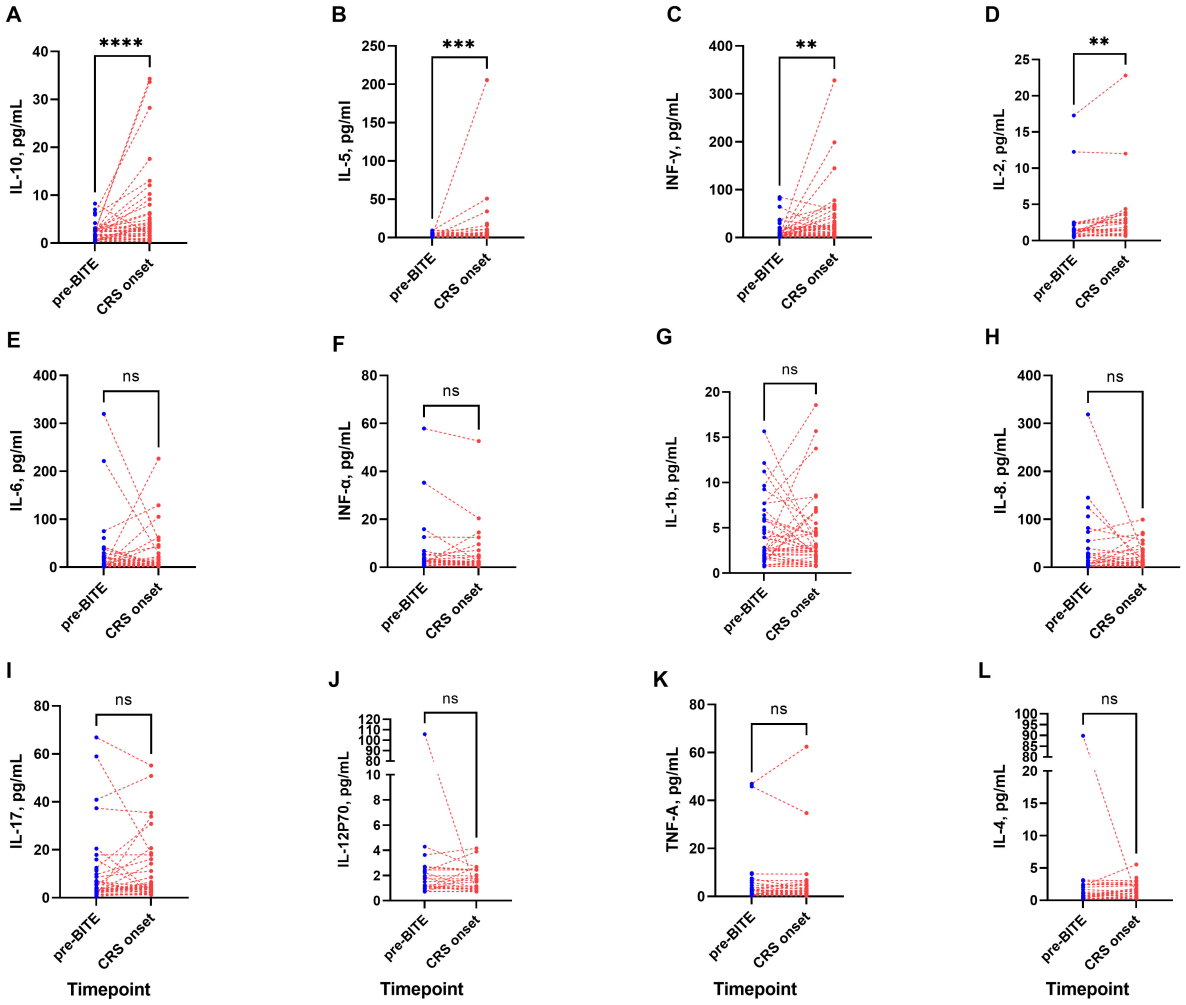
Cytokine dynamics during blinatumomab therapy. **(A-D)** IL-10, IL-5, IFN-γ, and IL-2 increased significantly at CRS-onset. **(E-H)** IL-6, IFN-α, IL-1β, and IL-8 levels remained stable during blinatumomab. **(I-L)** No significant alterations occurred in IL-17, IL-12p70, TNF-α, and IL-4. **P< 0.01; ***P< 0.001; ****P< 0.0001.

IL-10 increased from 2.4 (0.3–8.3) to 2.4 (0.3–34.3) pg/mL (P < 0.0001; **Figure 3A**). IL-5 rose from 2.7 (0.3–9.6) to 2.7 (0.5–205.6) pg/mL (P = 0.0006; **Figure 3B**). IFN-γ increased from 4.6 (1.0–84.5) to 12.3 (1.3–328.3) pg/mL (P = 0.003; **Figure 3C**). IL-2 rose from 2.4 (0.5–17.3) to 2.4 (0.7–22.8) pg/mL (P = 0.001; **Figure 3D**).

No significant changes were observed in IL-6, IL-8, IL-4, IL-1β, IL-12p70, IL-17, TNF-α, or IFN-α between pre-blinatumomab and CRS onset (**Figures 3E–L**).

### Overall survival and event free survival

In total, nine patients had NR after the first cycle of blinatumomab. During follow-up, four relapses and one death were documented. Among the relapses, three occurred in the R/R group and one in a CR-MRD^pos^ patient with subsequent MRD reversion to positivity. Of these, three were BM recurrences and one was a CNS relapse. Additionally, one CR-MRD^neg^ patient died from CNS-invasive aspergillosis.

**Figure 4:**
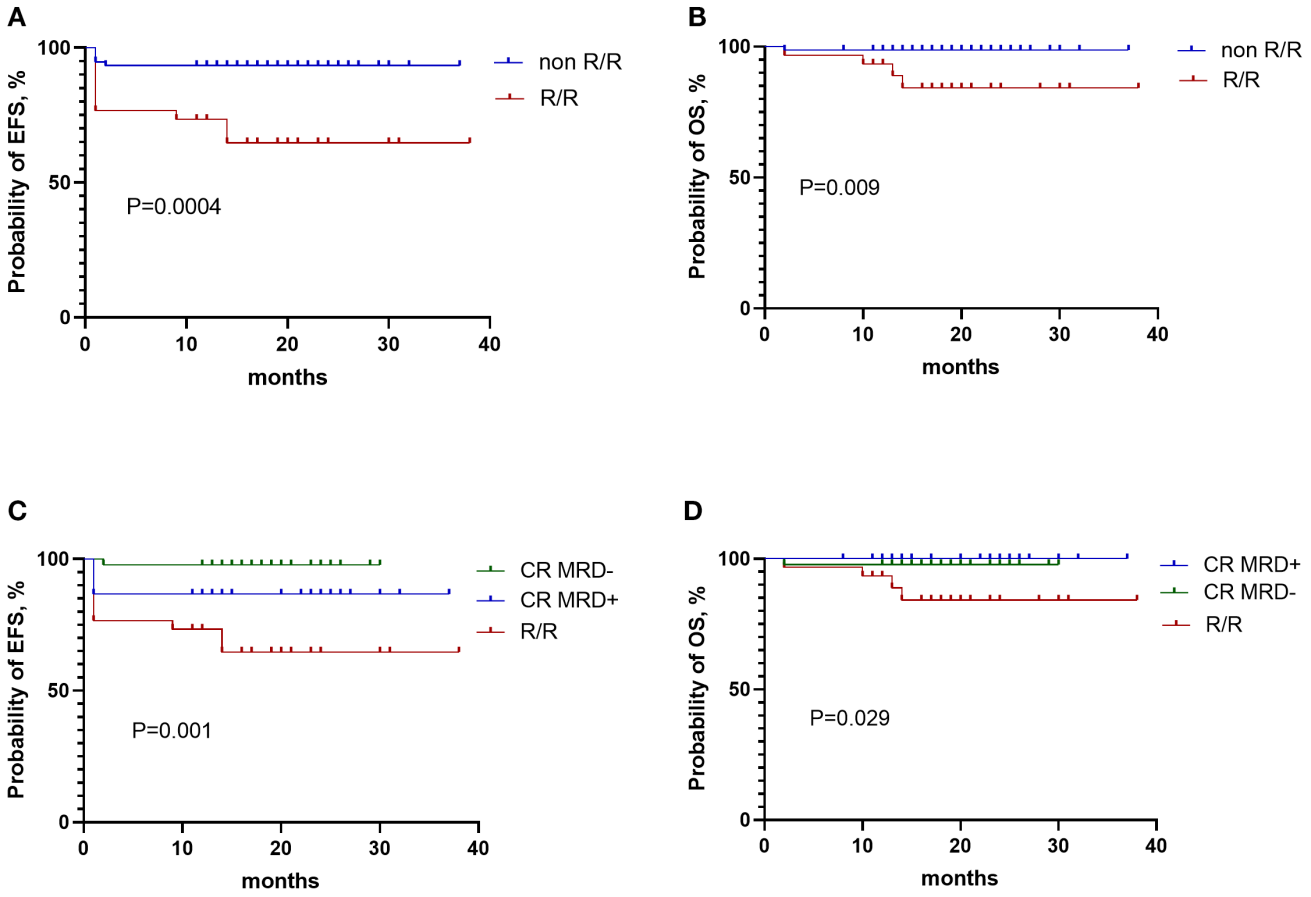
Survival outcomes of ALL patients receiving blinatumomab. **(A)** EFS in patients with and without R/R ALL. **(B)** OS in patients with and without R/R ALL. **(C)** EFS in patients with CR-MRD^neg^, CR-MRD^pos^, and R/R ALL. **(D)** OS in patients with CR-MRD^neg^, CR-MRD^pos^, and R/R ALL.

In patients with and without R/R status, the 1-year EFS rates were 73.3% ± 8.1% and 93.3% ± 2.9%, respectively (P = 0.0004; **Figure 4A**). The 1-year OS rates were 93.3% ± 4.6% and 98.6% ± 1.3%, respectively (P = 0.009; **Figure 4B**).

In patients with CR-MRD^neg^ and CR-MRD^pos^ status, the 1-year EFS rates were 97.8% ± 2.2% and 86.7% ± 6.2%, respectively (P = 0.001; **Figure 4C**). The 1-year OS rates were 97.8% ± 2.2% and 100%, respectively (P = 0.029; **Figure 4D**).

In patients with R/R and CR-MRD^pos^ status at initial classification, the 1-year EFS was 94.0% ± 3.4% for those achieving MRD negativity versus 10.0% ± 9.5% for those not achieving MRD negativity after blinatumomab (P < 0.0001). The corresponding 1-year OS rates were 100% and 78.8% ± 13.4% (P = 0.001).

In the R/R ALL subgroup, the 1-year EFS was 87.5% ± 6.8% for patients who achieved MRD negativity after blinatumomab therapy, compared with 16.7% ± 15.2% for those who remained MRD positive (P < 0.0001). The 1-year OS rates were 100% and 66.7% ± 19.2%, respectively (P = 0.002).

In the CR-MRDpos cohort, the 1-year EFS was 100% for patients who achieved MRD negativity versus 0% for those who remained MRD positive (P < 0.0001). The 1-year OS was 100% in both groups (P = 1.000).

### Risk factors of treatment failure

Among the 51 cases with CR-MRD^pos^ or R/R status, nine cases failed to respond. Univariable analysis revealed poor cytogenetics, BCR*-*ABL1 fusion, and low absolute B-cell count as risk factors for treatment failure (**Table 2**). Multivariable analysis using a Cox regression model further demonstrated that high MRD level (P = 0.014), BCR*-*ABL1 fusion (P = 0.065), and poor cytogenetics (P = 0.025) were independent risk factors.

**Table 2:** Factors for blinatumomab treatment response in B-cell ALL.

Features	CR, n=42	NR, n=9	P value
Gender, F/M, n	14/28	3/6	1.000
Age, median (range), mons	72.5 (6.0-208.0)	64.0 (5.0-152.0)	0.539
BM Blasts, median (range), %	0 (0-80)	1 (0-63)	0.345
MRD value, median (range), %	0.51 (0.004-62.69)	0.98 (0.012-63)	0.228
Risk			0.001
Poor cytogenetic, n (%)	8 (19.0)	7 (77.8)	
Good cytogenetic, n (%)	34 (81.0)	2 (22.2)	
Fusion gene			
KMT2Ar, n (%)	5 (11.9)	2 (22.2)	0.592
Non- KMT2Ar, n (%)	37 (88.1)	7 (77.8)	
ETV6/RUNX1, n (%)	5 (11.9)	0 (0)	0.571
Non-ETV6/RUNX1, n (%)	37(88.1)	9 (100)	
BCR/ABL1, n (%)	1 (2.4)	4 (44.4)	0.002
Non- BCR/ABL1, n (%)	41 (97.6)	5 (55.6)	
T and B cell subtype*			
Lymphocyte, median (range), 10^9^/L	0.96 (0.05-4.44)	0.19 (0.10-1.81)	0.270
CD3+ value, median (range), 10^9^/L	0.71 (0.03-3.79)	0.24 (0.08-1.67)	0.359
CD3+, %	78.59 (42.49-94.64)	85.80 (62.78-94.62)	0.443
CD4+, median (range), 10^9^/L	0.39 (0.01-1.12)	0.11 (0.03-0.72)	0.358
CD4+, %	33.24 (15.98-66.30)	38.25 (20.75-49.90)	0.696
CD8+, median (range), 10^9^/L	0.36 (0.01-2.39)	0.09 (0.02-0.94)	0.480
CD8+, %	34.19 (9.82-60.20)	34.72 (22.62-54.91)	0.856
CD19+ B cell, median (range), 10^9^/L	0.03 (0.00-1.12)	0.001 (0.00-0.02)	0.046
CD19+ B, %	3.01 (0.00-38.30)	0.27 (0.00-1.40)	0.041

CR, Complete remission; NR, no remission; MRD, minimal residual disease; BM, bone marrow; F/M, female/male; *defined as the value before blinatumomab treatment.

To evaluate the impact on MRD negativization, univariable analysis revealed poor cytogenetics (P = 0.0003), BCR*-*ABL1 fusion (P = 0.004), and low absolute B-cell count (P = 0.065) as risk factors (**Supplementary Table S3**). The Cox regression model showed that high MRD level (P = 0.014) and poor cytogenetics (P = 0.009) were independent risk factors.

In the initial cohort of 30 patients with R/R disease, 22 patients receiving bridging therapy demonstrated a CR rate of 86.4% (19/22), while the CR rate was 75.0% (6/8) in patients without bridging therapy (P = 0.589). Details of bridging therapy and response for R/R patients are presented in **Supplementary Table S4**. A subset of eight patients received bridging chemotherapy following induction therapy, with cyclophosphamide administered at doses of either 1,000 mg/m² or 300 mg/m². No statistically significant difference in CR rate was observed between patients receiving cyclophosphamide-containing bridging chemotherapy (87.5%, 7/8) and those who did not undergo lymphodepleting chemotherapy (81.8%, 18/22; *P* = 1.000; **Supplementary Table S4**).

### Adverse events

Each patient received one to four courses of blinatumomab, with a total of 125 cycles across the entire cohort. The most common AEs were CRS and hematologic toxicity. The incidence of severe CRS and ICANS in the R/R and CR-MRD^pos^ groups was comparable to that in the CR-MRD^neg^ group (3.9% vs. 0% and 0% vs. 5.4%, respectively; P = 0.146 and P = 0.399; **Table 3**).

**Table 3:** Adverse effects in ALL patients receiving blinatumomab.

Adverse events	Total, n	%	CR-MRD^neg^, n	%	R/r, CR-MRD^pos^, n	%	P value
CRS							0.146
G0	69	55.2	45	60.8	24	47.1	
G1-2	54	43.2	29	39.2	25	49.0	
G3-4	2	1.6	0	0	2	3.9	
ICANS							0.399
G0	119	95.2	69	93.2	50	98.0	
G1-2	2	1.6	1	1.4	1	2.0	
G3-4	4	3.2	4	5.4	0	0	
Infection	17	13.6	10	13.5	7	13.7	1.000
TLS	0	0	0	0	0	0	–
Neutropenia							0.004#
G0	62	51.7	41	55.4	21	45.7	
G1-2	26	21.7	9	12.2	17	37.0	
G3-4	32	26.7	24	32.4	8	17.4	
Thrombocytopenia							0.003*
G0	112	92.6	71	98.6	41	83.7	
G1-2	5	4.1	1	1.4	4	8.2	
G3-4	4	3.3	0	0	4	8.2	

ALL, acute lymphoblastic leukemia; MRD, minimal residual disease; R/R, relapsed/refractory; CR-MRD^pos^, complete remission with MRD positive; CR-MRD^neg^, complete remission with MRD negative; G, grade; TLS, tumor lysis syndrome; CRS, cytokine release syndrome; ICANS, Immune Effector Cell-Associated Neurotoxicity Syndrome; *analysis G0 with G1-4; # analysis G0 with G1–2 and G3-4.

Among the six patients who developed ICANS, the median age was 160 months, significantly older than that of patients without ICANS [160.5 (69–210) vs. 73 (5–212) months; P = 0.014]. Four patients underwent serum cytokine profiling both before and at ICANS onset, while three patients additionally received cerebrospinal fluid (CSF) cytokine profiling. During ICANS onset, CSF showed a white blood cell count of 0–40 cells/μL, albumin levels between 300–783 mg/L, and cytokine profiling in three patients revealed elevations of IL-5, IL-6, and IL-8, while IL-2, IL-10, and IFN-γ remained within reference ranges. Meanwhile, most serum cytokines showed no abnormal elevations; however, elevated IL-8 was observed. Detailed data are shown in **Table 4**.

**Table 4:** Cytokine levels in serum and cerebrospinal fluid during ICANS episodes.

Cytokine	IL-5, pg/mL			IL-2, pg/mL			IL-6, pg/mL			IL-10, pg/mL			IFN-γ, pg/mL			IL-8, pg/mL		
	Pre	35.8Onset	35.8CSF	35.8Pre	35.8Onset	35.8CSF	35.8Pre	35.8Onset	35.8CSF	35.8Pre	35.8Onset	35.8CSF	35.8Pre	35.8Onset	35.8CSF	35.8Pre	35.8Onset	CSF
Case 1	2.44	2.44	37.03	2.44	2.44	2.74	3.42	2.98	22.24	2.44	2.44	2.44	3.54	3.89	3.65	3.6	15.33	814.94
Case 2	2.44	2.44	–	2.44	2.44	–	37.99	3.3	–	2.44	2.44	–	8.81	2.44	–	2.44	41.9	–
Case 3	0.51	0.87	1.55	0.65	0.65	1.49	0.48	1.32	6.29	0.32	0.32	0.32	1.49	3.22	1.49	1.93	14.1	227.66
Case 4	5.27	0.77	1.65	2.53	0.65	0.89	2.45	1.44	5.29	0.93	1.27	0.32	1.85	3.11	1.49	7.83	12.3	112.3

ICANS, Immune Effector Cell-Associated Neurotoxicity Syndrome; Pre, Serum concentration pre-blinatumomab; Onset, serum concentration at ICANS onset; CSF, CSF concentration at ICANS onset.

Five of six patients with ICANS underwent T-cell subset analysis. A more pronounced inversion of the CD4+/CD8+ ratio—particularly with higher CD8+ proportions, together with lower absolute B-cell counts and reduced B-cell percentages—was implicated in a higher risk of ICANS (**Supplementary Table S5**).

Severe neutropenia occurred more frequently in the CR-MRD^neg^ group compared with the high-MRD group (32.4% vs. 17.4%; P = 0.003). Thrombocytopenia was more common in the R/R plus MRD^pos^ group than in the CR-MRD^neg^ group (16.4% vs. 1.4%; P = 0.003).

## Discussion

Blinatumomab, the first bispecific T-cell engager approved for R/R B-ALL, has demonstrated remarkable clinical outcomes across multiple cohorts. In our study, we observed a high overall response rate of 82.4% in B-ALL, including both R/R and MRD^pos^ cases. The 1-year EFS and OS for R/R patients were 73.3% and 93.3%, respectively. These favorable outcomes may be attributed to the robust response to blinatumomab, often followed by HSCT or CAR-T therapy. Compared with standard salvage chemotherapy, patients with R/R B-ALL treated with blinatumomab exhibited significantly improved OS (11, 12). In the Children’s Oncology Group AALL1331 study, patients with low-risk first relapse of B-ALL were randomized to receive either chemotherapy cycles or chemotherapy intercalated with three blocks of blinatumomab. The 4-year disease-free survival (DFS)/OS for 255 patients were 61.2% and 90.4% in the blinatumomab group, compared with 49.5% and 79.6% in the chemotherapy group (P = 0.089 and 0.11) (13). In another study evaluating blinatumomab as consolidation, children with high-risk first-relapse B-ALL were randomized to receive one cycle of blinatumomab or a third course of consolidation chemotherapy prior to HSCT. A higher MRD remission rate was observed in the blinatumomab group compared with chemotherapy (90% [44/49] *vs.* 54% [26/48]), along with improved EFS (14). In a randomized phase 3 clinical trial, patients received either two cycles of blinatumomab or two cycles of multiagent chemotherapy after reinduction chemotherapy, followed by transplantation. With a median follow-up of 2.9 years, 2-year DFS and OS were superior in the blinatumomab group compared with the chemotherapy group (54.4% *vs.* 39.0%, P = 0.03; 71.3% *vs.* 58.4%, P = 0.02) (15).

Persistence or recurrence of CR-MRD^pos^ was mainly attributed to delayed MRD clearance and subsequent re-emergence, primarily due to adverse cytogenetic profiles and delays in chemotherapy administration. In our study, these patients received blinatumomab as a preemptive intervention. Although CR-MRD^pos^ patients demonstrated inferior 1-year EFS compared with CR-MRD^neg^ patients (86.7% *vs.* 97.8%), their outcomes were better than those of R/R patients. Notably, the 1-year OS for CR-MRD^pos^ patients was 100%, higher than the 97.7% observed in CR-MRD^neg^ patients. These findings indicate that blinatumomab was both safe and effective in patients with chemotherapy intolerance or resistance. In a matched cohort study evaluating blinatumomab as an alternative to intensive post-remission chemotherapy for chemotherapy-intolerant or resistant patients, comparable 2-year EFS and OS rates were seen between the blinatumomab-treated cohort (n = 80) and conventional chemotherapy controls (n = 192): 95% vs. 90% and 97% vs. 94%, respectively (16).

The mechanisms underlying blinatumomab resistance remain incompletely understood. Our study identified adverse cytogenetics, BCR*-*ABL1 fusion, and low absolute CD19+ B-cell counts as significant predictors of treatment failure. Furthermore, elevated MRD burden, BCR*-*ABL1 positivity, and high-risk cytogenetic profiles emerged as independent risk factors. Previous studies have shown that lower tumor burden is associated with higher CR rates (14, 17, 18), which in turn influence DFS and OS (18). Consistent with prior findings, our data confirm the association between elevated MRD levels and suboptimal treatment response (19). Our findings also suggest that low absolute CD19+ B-cell count contributes to blinatumomab resistance. In line with earlier investigations, pre-blinatumomab absolute lymphocyte count (ALC) and the MRD/ALC ratio were associated with MRD response. Analysis revealed prognostic associations for pre-blinatumomab MRD level, ALC, MRD/ALC ratio, and post-blinatumomab MRD remission with OS and EFS (19). Among the poor cytogenetics, BCR*-*ABL1 fusion was the main predictor of blinatumomab resistance in our cohort.

Outcomes in patients with Philadelphia chromosome (Ph)–positive ALL have improved with the use of TKIs. A chemotherapy-free induction and consolidation regimen combining dasatinib and blinatumomab reported a high induction CR rate of 98%. The molecular response at the end of dasatinib induction therapy (29%) increased to 60% after two cycles of blinatumomab (20). Nevertheless, its application remains rare in the pediatric setting.

The MRD monitoring in this research was performed using FCM and applied to all cases, with a sensitivity of 0.01%. The MRD cut-off was appropriate according to the recommendation of the 2024 European LeukemiaNet (ELN) (21). FCM can be applied to most ALL cases (>90%), and the results are promptly available. Molecular MRD monitoring of fusion genes (e.g., BCR*-*ABL1) has a sensitivity of around 0.01%. However, its accuracy is hampered by the variability in the number of RNA transcripts in leukemic cells. In extremely low-burden cases, novel techniques such as digital droplet PCR and next-generation sequencing (NGS) could be used. The use of qPCR measurement of clonal immunoglobulin/T-cell receptor (IG/TR) in Ph’- ALL could be more precise, as recommended in ELN 2024. Despite the promising efficacy of blinatumomab, its impact on host immune cell dynamics remains incompletely understood. T-cell activation may play a critical role in modulating blinatumomab responsiveness. To address this, we systematically characterized the immune cell repertoire at baseline, throughout treatment, and post-therapy. Our immunophenotyping data demonstrated significant temporal expansion of CD3+, CD8+, CD4+ T cells, and NK cells by day 14, with sustained elevation through days 21–28, consistent with prior observations (22). Recent studies have further elucidated blinatumomab-mediated modulation of peripheral blood T-cell subset distribution during therapy (20, 23–25). Circulating T cells were found to decrease within the first day of infusion and then recover to baseline after approximately one week, likely due to increased T-cell adhesion to blood vessel endothelium (25). During the T-cell activation phase, we observed near-complete depletion of circulating B lymphocytes across nearly all cases. *In vitro* coculture experiments have shown that blinatumomab can induce redirected lysis of CD19+ B lymphocytes and malignant B-cell lines by previously resting peripheral T cells (26).

Notably, blinatumomab exhibited differential immunomodulatory effects across distinct patient subgroups, with marked variations observed between R/R and CR-MRD^pos^ patients compared with CR-MRD^neg^ (chemotherapy-intolerant) patients. Analysis revealed significantly greater activation of CD3+ and CD4+ T cells in the R/R and CR-MRD^pos^ cohorts relative to the CR-MRD^neg^ group. Our cohort showed that the proportion of T cells was not related to response. An interesting case series reported by Duminuco indicated that a higher proportion of baseline T lymphocytes achieved MRD negativity more frequently, though without statistical significance (P = 0.06) (27). Concomitantly, more profound depletion of B cells was seen in the R/R and CR-MRD^pos^ groups, likely attributable to enhanced CD19-directed cytolysis of malignant B-cell populations by activated T cells. Following blinatumomab, decreased IgG levels were noted among the tested patients. Taken together, these findings indicate that blinatumomab induces transient but significant immunophenotypic remodeling, characterized by preferential expansion of CD3+ and CD4+ T-cell compartments. However, no statistically significant differences were observed in NK cell counts, total lymphocytes, or CD8+ T-cell populations at the end of the cycle compared with pretreatment levels.

Relapses occurred in four patients during follow-up, with three in the R/R group and one in the CR-MRD^pos^ group. Two cases developed CD19-negative relapses, a well-documented mechanism of blinatumomab resistance, with reported incidence rates ranging from 8% to 35% in clinical studies (28–30). The relapse pattern included isolated CNS involvement in one patient (25%). Two cases represented early treatment failure, with relapses occurring within one month of blinatumomab initiation. The remaining two patients experienced late relapse at nine and 14 months post-therapy, respectively.

The safety profile observed in this study was consistent with the AE spectrum reported in prior clinical trials. No treatment-related mortality was reported. CRS of grade ≥3 severity occurred in two patients (1.6%), exclusively within the R/R cohort. Neurological events of grade ≥3 were documented in four patients, all in the CR-MRD^neg^ group, with older age identified as a significant risk factor for neurotoxicity. The incidence of grade ≥3 CRS and neurotoxicity was 1.6% and 3.2%, aligning with published safety data reporting severe CRS and neurotoxicity incidences of 1–3.1% and 1–7%, respectively (13, 15, 31). Grade 3–4 neutropenia was more common in the CR-MRD^neg^ population than in R/R patients, while any-grade thrombocytopenia was more frequently observed in the combined R/R and CR-MRD^pos^ groups compared with CR-MRD^neg^ cases. Overall, the incidence of severe AEs remained low, and no blinatumomab-related death was observed.

Blinatumomab demonstrated encouraging results in children with R/R ALL and MRD-positive disease. Notably, it emerged as a particularly valuable therapeutic option for chemotherapy-intolerant patients or those with severe concurrent infections, serving as an effective bridging therapy to maintain durable MRD negativity. Treatment failure occurred in approximately 10–15% of cases, with growing evidence suggesting that intrinsic disease biology, including specific cytogenetic abnormalities and immunophenotypic profiles, significantly influences therapeutic response. Comprehensive pretreatment evaluation incorporating high-risk genetic markers and quantitative CD19+ B-cell assessment may facilitate more precise identification of therapy-sensitive and therapy-resistant subgroups, potentially informing risk-adapted treatment strategies.

## Data Availability

The original contributions presented in the study are included in the article/**Supplementary Material**. Further inquiries can be directed to the corresponding authors.
