# Supplementary material for: Blinatumomab demonstrates MRD eradication in MRD-positive/chemotherapy-delayed pediatric B-ALL and high response in relapsed/refractory cases: a multicenter cohort study
Source: Front Immunol. 2025 Sep 18;16:1607138. doi: 10.3389/fimmu.2025.1607138 (PMC12488715; doi:10.3389/fimmu.2025.1607138)
Supplement: Supplementary file 1 [file Table1.docx]

Supplemental table 1 T cell activation following blinatumomab therapy.

| T cell subtype | Baseline | Post blinatumomab | P value |
| --- | --- | --- | --- |
| CD3+, median (range),10^9^/L | 0.96 (0.03–3.79) | 1.13 (0.26–7.74) | 0.016 |
| 14-day infusing | 1.02 (0.10–3.79) | 1.15 (0.26–6.98) | 0.047 |
| 21/28-day infusing | 0.80 (0.03–1.86) | 1.01 (0.33–7.74) | 0.033 |
| CD3+, % | 85.44 (42.49–99.08) | 86.72 (56.97–97.73) | 0.008 |
| 14-day infusing | 85.44 (42.49-97.54) | 85.02 (56.97-97.73) | 0.097 |
| 21/28-day infusing | 82.18 (66.61-99.08) | 92.00 (78.99-97.20) | 0.016 |
| CD4+, median (range), 10^9^/L | 0.35 (0.01–1.39) | 0.47 (0.07–2.94) | 0.005 |
| 14-day infusing | 0.35 (0.07–1.39) | 0.47 (0.07–2.94) | 0.026 |
| 21/28-day infusing | 0.42 (0.01–0.72) | 0.47 (0.12–1.25) | 0.092 |
| CD4+, % | 32.78 (17.25–66.30) | 33.78 (15.28–72.00) | 0.025 |
| 14-day infusing | 31.32 (17.25-61.50) | 33.19 (15.28-63.13) | 0.048 |
| 21/28-day infusing | 38.55 (21.16-66.30) | 39.55 (15.36-72.00) | 0.519 |
| CD8+, median (range), 10^9^/L | 0.41 (0.01–2.39) | 0.56 (0.07–6.07) | 0.006 |
| 14-day infusing | 0.41 (0.03–2.39) | 0.61 (0.07–3.85) | 0.011 |
| 21/28-day infusing | 0.45 (0.10–6.07) | 0.32 (0.01–1.03) | 0.470 |
| CD8+, % | 40.95 (9.82–64.41) | 41.95 (13.05–74.78) | 0.054 |
| 14-day infusing | 40.98 (11.92-64.41) | 42.73 (13.05-67.56) | 0.624 |
| 21/28-day infusing | 38.36 (9.82-52.43) | 40.63 (15.77-74.78) | 0.266 |
| CD16+CD56+/CD3- median (range), 10^9^/L | 0.12 (0.001–0.63) | 0.15 (0.03–0.74) | 0.024 |
| 14-day infusing | 0.12 (0.001-0.63) | 0.18 (0.03-0.74) | 0.013 |
| 21/28-day infusing | 0.10 (0.002–0.51) | 0.08 (0.03–0.47) | 0.850 |
| CD16+CD56+/CD3-, % | 11.69 (0.79-41.04) | 12.60 (1.85-46.24) | 0.630 |
| 14-day infusing | 11.90 (1.87-41.04) | 14.49 (1.85-46.24) | 0.173 |
| 21/28-day infusing | 10.40 (0.79-21.63) | 7.69 (2.40-20.51) | 0.110 |

Supplemental table 2 T cell activation in R/R+MRD^pos^ and CR-MRD^neg^ groups.

| Activation magnitude* | R/R+MRD ^pos^ | MRD^neg^ | P value |
| --- | --- | --- | --- |
| CD3+ T cells, median (range), 10^9^/L | 0.57 (-1.08–6.07) | 0.12 (-1.83–3.34) | 0.047 |
| CD4+ cells, median (range), 10^9^/L | 0.24 (-0.55–1.81) | 0.03 (-0.73–1.19) | 0.039 |
| CD8+ cells, median (range), 10^9^/L | 0.16 (-0.48–5.21) | 0.11 (-0.58–1.33) | 0.174 |
| B cells, median (range), 10^9^/L | -0.03 (-1.12–0.00) | -0.004 (-0.35–0.08) | 0.017 |
| NK cell, median (range), 10^9^/L | 0.01 (-0.10–0.53) | 0.03 (-0.32–0.57) | 0.844 |
| Lymphocytes, median (range), 10^9^/L | 0.30 (-1.56–6.61) | 0.06 (-1.25–3.55) | 0.107 |
| CD4+/CD8+ ratio, median (range) | 0.07 (-3.90–1.54) | 0.01 (-1.23–1.01) | 0.406 |
| Neutrophils, median (range), 10^9^/L | -0.11 (-5.61–4.10) | -0.09 (-15.61–3.41) | 0.788 |
| Platelets, median (range), 10^9^/L | -5.00 (-195.0–607.0) | -10.00 (-313.0–357.0) | 0.219 |

* Defined as the difference between post-blinatumomab and baseline (pre-treatment) measurement.

Supplemental Table 3 The impact of MRD negativization in patients with R/R and CR-MRD positive.

| feature | MRD negativization, n=41 | MRD persistence, n=10 | P value |
| --- | --- | --- | --- |
| Reinduction strategy |  |  | 0.702 |
| Bridge-to chemotherapy, n (%) | 10 (24.4) | 3 (30.0) |  |
| Upfront blinatumomab, n (%) | 31 (74.6) | 7 (70.0) |  |
| Disease status |  |  | 0.669 |
| R/R, n (%) | 8 (19.5) | 3 (30.0) |  |
| CR-MRD^pos^, n (%) | 33 (80.5) | 7 (70.0) |  |
| CD3+, median (range) 10^9^/L | 0.75 (0.03-3.79) | 0.38 (0.08-1.67) | 0.314 |
| CD3+, median (range), % | 80.41 (42.49-94.64) | 82.52 (62.78-94.62) | 0.745 |
| B cell, median (range), 10^9^/L | 0.03 (0-1.12) | 0.002 (0.00-0.02) | 0.065 |
| B19+, median (range), % | 2.98 (0.00-38.30) | 0.71 (0.00-3.04) | 0.085 |
| Risk |  |  | 0.0003 |
| Poor cytogenetic, n (%) | 7 (17.1) | 8 (80.0) |  |
| Good cytogenetic, n (%) | 34 (82.9) | 2 (20.0) |  |
| BCR/ABL1 fusion |  |  | 0.004 |
| yes, n (%) | 1 (2.4) | 4 (40.0) |  |
| no, n (%) | 40 (97.6) | 6 (60.0) |  |
| MRD level, median (range), % | 0.50 (0.004-62.69) | 1.66 (0.01-63.0) | 0.123 |

Supplemental table 4 Bridging chemotherapy and response in 30 patients with R/R disease.

| Pt | Disease status | Re-induction* | Bridging therapy (cyclophosphamide mg/m^2^) # | BM Blasts pre- blinatumomab, % | BM MRD pre- blinatumomab, % | Grouping | Response |
| --- | --- | --- | --- | --- | --- | --- | --- |
| 1 | induction failure | yes | 1000 | 0 | 15.16 | CR-MRD^pos^ | NR |
| 2 | induction failure | no | 0 | 20 | 46.87 | R/R | NR |
| 3 | induction failure | yes | 0 | 0 | 0.5 | CR-MRD^pos^ | NR |
| 4 | induction failure | yes | 1000 | 5 | 3 | R/R | CR |
| 5 | induction failure | no | 0 | 22 | 16.28 | R/R | CR |
| 6 | induction failure | no | 0 | 41 | 31.6 | R/R | CR |
| 7 | induction failure | no | 0 | 80 | 62.69 | R/R | CR |
| 8 | Relapse | yes | 1000 | 0 | 0.007 | CR-MRD^pos^ | CR |
| 9 | Relapse | yes | 0 | 0 | ＜0.01 | CR-MRD^neg^ | CR-MRD^neg^ |
| 10 | Relapse | no | 0 | 9.5 | 6.38 | R/R | CR |
| 11 | Relapse | yes | 0 | 0 | ＜0.01 | CR-MRD^neg^ | CR-MRD^neg^ |
| 12 | Relapse | no | 0 | 24.5 | 25.2 | R/R | CR |
| 13 | Relapse | yes | 1000 | 0 | ＜0.01 | CR-MRD^neg^ | CR-MRD^neg^ |
| 14 | Relapse | yes | 0 | 5 | 5.5 | R/R | CR |
| 15 | Relapse | no | 0 | 63 | 63 | R/R | NR |
| 16 | Relapse | yes | 0 | 2.5 | 3.5 | CR-MRD^pos^ | CR |
| 17 | Relapse | yes | 0 | 2 | ＜0.01 | CR-MRD^neg^ | CR-MRD^neg^ |
| 18 | Relapse | yes | 0 | 0.5 | ＜0.01 | CR-MRD^neg^ | CR-MRD^neg^ |
| 19 | Relapse | yes | 1000 | 0.5 | ＜0.01 | CR-MRD^neg^ | CR-MRD^neg^ |
| 20 | Relapse | no | 0 | 20 | 20 | R/R | CR |
| 21 | Relapse | yes | 0 | 5 | 5 | R/R | CR |
| 22 | Relapse | yes | 0 | 1 | 0.01 | CR-MRD^pos^ | CR |
| 23 | Relapse | yes | 300 | 0.5 | ＜0.01 | CR-MRD^neg^ | CR-MRD^neg^ |
| 24 | Relapse | yes | 1000 | 0 | ＜0.01 | CR-MRD^neg^ | CR-MRD^neg^ |
| 25 | Relapse | yes | 0 | 0 | ＜0.01 | CR-MRD^neg^ | CR-MRD^neg^ |
| 26 | Relapse | yes | 0 | 0 | 0.03 | CR-MRD^pos^ | CR |
| 27 | Relapse | yes | 300 | 0 | 0.03 | CR-MRD^pos^ | CR |
| 28 | Relapse | yes | 0 | 0 | 0.04 | CR-MRD^pos^ | CR |
| 29 | Relapse | yes | 0 | 1 | 2.33 | CR-MRD^pos^ | NR |
| 30 | Relapse | yes | 0 | 1.8 | 1.84 | CR-MRD^pos^ | CR |

*The reinduction regimen included: dexamethasone 6 mg/m^2^ on days 1-4; vincristine 1.5 mg/m^2^, on days 5, 12, 19, 27; prednisone 45 mg/m^2^ on days 5-28; daunorubicin 25 mg/m^2^ on days 5, 12; Peg-Asparaginase 2000 U/m^2^ on days 6, 26. For patients with Ph’+, tyrosine kinase inhibitor was combined, with dasatinib 80 mg/m^2^ preferred.

#Bridging chemotherapy included induction chemotherapy or continued consolidation chemotherapy (cyclophosphamide 1000 mg/m^2^ on day 1; cytarabine 50 mg/m^2^ on days 1-7; mercaptopurine 40 mg/m^2^ on days 1-7). In some cases, a subset of patients received maintenance therapy comprising cyclophosphamide 300 mg/m² and cytarabine 300 mg/m² prior to blinatumomab administration.

Supplemental Table 5 T cell subtype in patients with and without ICANS.

| Features* | ICANS | Non-ICANS | P value |
| --- | --- | --- | --- |
| Lymphocyte, median (range), 10^9^/L | 0.91 (0.34-3.81) | 0.99 (0.05-6.25) | 0.877 |
| CD3+, median (range), 10^9^/L | 1.10 (0.41-3.27) | 0.77 (0.03-4.29) | 0.516 |
| CD4+, median (range), 10^9^/L | 0.25 (0.13-1.20) | 0.34 (0.01-2.01) | 0.783 |
| CD8+ median (range), 10^9^/L | 0.86 (0.27-1.97) | 0.39 (0.01-2.39) | 0.142 |
| CD16+56+/CD3-, median (range), 10^9^/L | 0.17 (0.04-0.50) | 0.10 (0.84-1.22) | 0.299 |
| CD19+ B cell, median (range), 10^9^/L | 0.0004 (0.00-0.02) | 0.01 (0.00-1.43) | 0.027 |
| CD4+/CD8+ | 0.47 (0.27-0.80) | 0.90 (0.29-4.92) | 0.004 |
| CD3+, % | 90.70 (81.61-99.30) | 85.07 (42.49-99.08) | 0.071 |
| CD4+, % | 28.10 (17.83-43.58) | 33.89 (15.98-66.30) | 0.131 |
| CD8+, % | 60.20 (49.79-65.80) | 39.45 (9.82-64.41) | 0.0002 |
| CD19+, % | 0.03 (0.00-0.89) | 1.54 (0.00-38.30） | 0.010 |
| CD16+56+/CD3-, % | 11.26 (6.21-17.67) | 11.78 (0.79-63.39) | 0.848 |

*, T and B cell subtype was defined as the value before blinatumomab treatment.
